# Survey of Early-Diverging Lineages of Fungi Reveals Abundant and Diverse Mycoviruses

**DOI:** 10.1128/mBio.02027-20

**Published:** 2020-09-08

**Authors:** J. M. Myers, A. E. Bonds, R. A. Clemons, N. A. Thapa, D. R. Simmons, D. Carter-House, J. Ortanez, P. Liu, A. Miralles-Durán, A. Desirò, J. E. Longcore, G. Bonito, J. E. Stajich, J. W. Spatafora, Y. Chang, L. M. Corrochano, A. Gryganskyi, I. V. Grigoriev, T. Y. James

**Affiliations:** a University of Michigan, Department of Ecology and Evolutionary Biology, Ann Arbor, Michigan, USA; b University of California—Riverside, Department of Microbiology and Plant Pathology, Riverside, California, USA; c University of Seville, Department of Genetics, Seville, Spain; d Michigan State University, Department of Plant Pathology, East Lansing, Michigan, USA; e University of Maine, School of Biology and Ecology, Orono, Maine, USA; f Oregon State University, Department of Botany and Plant Pathology, Corvalis, Oregon, USA; g L. F. Lambert Spawn Co., Coatesville, Pennsylvania, USA; h U.S. Department of Energy Joint Genome Institute, Lawrence Berkeley National Laboratory, Berkeley, California, USA; i University of California—Berkeley, Department of Plant and Microbial Biology, Berkeley, California, USA; University of California, Berkeley

**Keywords:** mycovirus, dsRNA virus, Chytridiomycota, Blastocladiomycota, Neocallimastigomycota, Zoopagomycota, Mucoromycota, double-stranded RNA virus, mycoviruses

## Abstract

Mycoviruses are widespread and purportedly common throughout the fungal kingdom, although most are known from hosts in the two most recently diverged phyla, Ascomycota and Basidiomycota, together called Dikarya. To augment our knowledge of mycovirus prevalence and diversity in underexplored fungi, we conducted a large-scale survey of fungi in the earlier-diverging lineages, using both culture-based and transcriptome-mining approaches to search for RNA viruses. In total, 21.6% of 333 isolates were positive for RNA mycoviruses. This is a greater proportion than expected based on previous taxonomically broad mycovirus surveys and is suggestive of a strong phylogenetic component to mycoviral infection. Our newly found viral sequences are diverse, composed of double-stranded RNA, positive-sense single-stranded RNA (ssRNA), and negative-sense ssRNA genomes and include novel lineages lacking representation in the public databases. These identified viruses could be classified into 2 orders, 5 families, and 5 genera; however, half of the viruses remain taxonomically unassigned. Further, we identified a lineage of virus-like sequences in the genomes of members of Phycomycetaceae and Mortierellales that appear to be novel genes derived from integration of a viral RNA-dependent RNA polymerase gene. The two screening methods largely agreed in their detection of viruses; thus, we suggest that the culture-based assay is a cost-effective means to quickly assess whether a laboratory culture is virally infected. This study used culture collections and publicly available transcriptomes to demonstrate that mycoviruses are abundant in laboratory cultures of early-diverging fungal lineages. The function and diversity of mycoviruses found here will help guide future studies into mycovirus origins and ecological functions.

## Abstract (precis)

Viruses are key drivers of evolution and ecosystem function and are increasingly recognized as symbionts of fungi. Fungi in early-diverging lineages are widespread, ecologically important, and comprise the majority of the phylogenetic diversity of the kingdom. Viruses infecting early-diverging lineages of fungi have been almost entirely unstudied. In this study, we screened fungi for viruses by two alternative approaches: a classic culture-based method and by transcriptome-mining. The results of our large-scale survey demonstrate that early-diverging lineages have higher infection rates than have been previously reported in other fungal taxa and that laboratory strains worldwide are host to infections, the implications of which are unknown. The function and diversity of mycoviruses found in these basal fungal lineages will help guide future studies into mycovirus origins and their evolutionary ramifications and ecological impacts.

## INTRODUCTION

**FIG 1:**
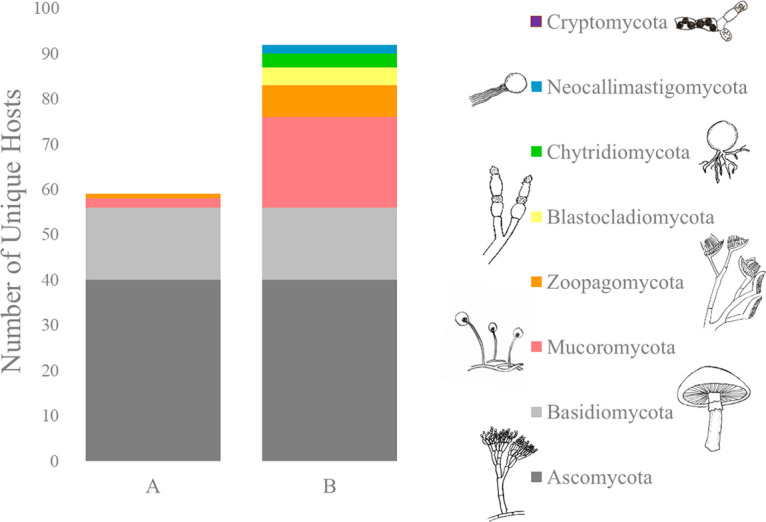
Bar plot showing the number of unique hosts of exogenous mycoviruses, per phylum, as represented in GenBank before this study (A) and with the data from this study added (B).

Fungal viruses (mycoviruses) have been reported from all major fungal taxonomic groups (1, 2). However, the overwhelming majority of mycoviruses have been identified in hosts belonging to just two phyla—Ascomycota and Basidiomycota (known together as “Dikarya”)—though the kingdom Fungi is comprised of at least eight phyla (3–5) (Fig. 1). Known mycovirus infections in non-Dikarya are limited to an early report of virus-like particles from ultrastructure studies of *Allomyces arbusculus* (6) and sequence-based identification in Rhizopus oryzae (7), Rhizopus microsporus (8), six isolates of arbuscular mycorrhizal fungi (9, 10), Umbelopsis ramanniana (11), and Entomophthora muscae (12, 13). While it has become clear that mycoviruses are widespread, disproportionate sampling across the fungal kingdom has resulted in an incomplete understanding of mycovirus diversity and prevalence. By sampling unexplored and phylogenetically diverse fungal lineages, we predicted to find equally diverse viruses that could empower meaningful inquiries into the origins and ecological functions of mycoviruses.

**FIG 2:**
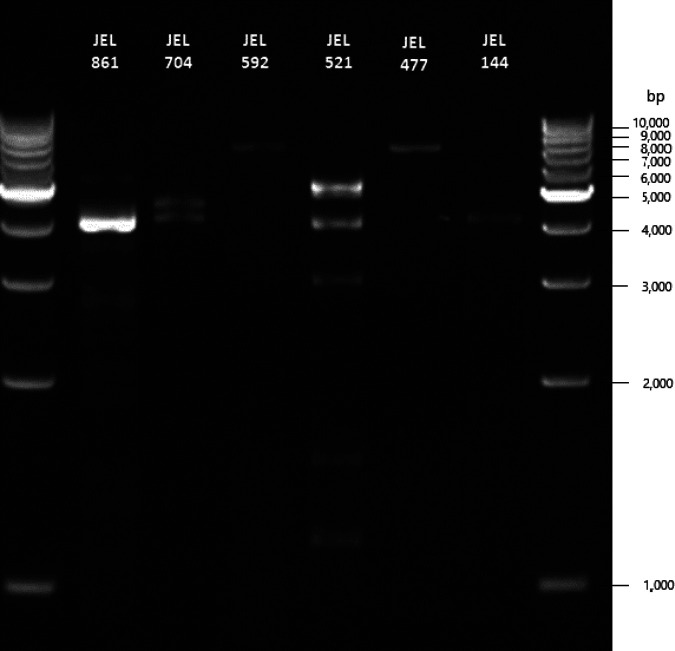
Agarose-gel electrophoresis image of the purified dsRNAs of six isolates of *Cladochytrium* sp. with varied banding patterns, flanked by 1-kb ladders. The first sample (lane 2) shows dsRNA of *Cladochytrium* sp. JEL861, which was sequenced in this study.

The conventional approach to mycovirus discovery exploits the observation that double-stranded RNA (dsRNA) genomes predominate among known mycoviruses (1). The most common method uses cellulose chromatography to purify dsRNA from total RNA extracts and effectively isolate mycoviruses with dsRNA genomes and the dsRNA replicative intermediates of single-stranded RNA (ssRNA) genomes (14). This approach is quick and inexpensive; however, its exclusive use ignores DNA viruses and reinforces the bias that mycoviral genomes are predominantly composed of RNA. Furthermore, viral RNA is detected visually by gel electrophoresis (see Fig. 2), which could result in false negatives in instances of low-titer infections. To our knowledge, estimates of the proportion of false negatives based on chromatography have not been reported. We tested the relative accuracy of cellulose chromatography for identifying fungal isolates with viral infection and compared the results to more indirect sequence-based alternative approaches.

*In silico* approaches to mycovirus discovery have become more common (15–17). These approaches search transcriptomic data for sequences similar to RNA-dependent RNA polymerase (RdRp), which is the only conserved gene among RNA viruses and is diagnostic of infection. Conceptually, these approaches should be more sensitive to low titer viral infections because they do not rely on gel visualization and high transcriptome sequencing depth is readily achieved. Indeed, RNA sequencing has revealed cryptic coinfection in isolates previously thought to be singularly infected (18) (J. Myers, unpublished data). As sequencing costs have dropped, the availability of transcriptome (RNA) sequencing data has grown, which presents a novel opportunity to probe existing data sets to characterize viruses for the first time. Gilbert et al. (17) developed a data-mining pipeline for mycovirus identification, applied it to public transcriptomes of fungi in the subphylum Pezizomycotina, and discovered 52 novel mycoviruses, demonstrating the utility of this approach. However, such approaches have yet to be applied widely to non-Dikarya lineages.

Our primary goal was to improve understanding of prevalence and sequence diversity of RNA mycoviruses in the early-diverging lineages of Fungi, specifically Mucoromycota, Zoopagomycota, Chytridiomycota, Blastocladiomycota, Neocallimastigomycota, and Cryptomycota/Rozellomycota. We screened early-diverging lineages with both a culture-based chromatography approach (“*in vitro*”) and a transcriptome data-mining approach (“*in silico*”). When possible, we compared methods by screening the same isolates used for transcriptome-generation with the *in vitro* method. In total, we screened 333 hosts, of which 72 (21.6%) harbored viruses. These results demonstrate that mycoviruses are abundant in the early-diverging lineages of the fungal kingdom, including in research laboratory cultures around the world.

## RESULTS AND DISCUSSION

### Viral prevalence.

Through deep-sequencing approaches, recent studies have revealed the abundance of viruses in pathogenic fungal species, marine environments, and soils (15, 19, 20). We build on this momentum by exploring viruses hosted in species from the deep branches of kingdom Fungi. In doing so, we more completely characterized the abundance of viruses across the kingdom and uncovered novel viral sequence diversity linked to specific fungal hosts. We determined the first sequences of mycoviruses in the particularly under-explored phyla Blastocladiomycota, Neocallimastigomycota, and Chytridiomycota (Fig. 1). In total, we screened 333 fungi spanning six phyla by either or both *in vitro* (cellulose chromatography) and *in silico* (transcriptome-mining) methods (see Table S1 in the supplemental material) and found one or more viruses in 72 isolates (21.6%), 65 of them not previously known as mycoviral hosts (Fig. 1). Of the 36 hosts for which we obtained viral sequence data—either by assembling from host transcriptomes or direct sequencing—we generated 154 complete or partial viral sequences which, using the criteria described above, we conservatively reduced to 85 unique viruses.

10.1128/mBio.02027-20.1 TABLE S1 Collection information and viral screening results for all isolates screened by both culture-based and transcriptome-mining approaches. Asterisks denote viral positives determined to be endogenous in the host genome. Download Table S1, XLSX file, 0.03 MB. Copyright © 2020 Myers et al. 2020 Myers et al. This content is distributed under the terms of the Creative Commons Attribution 4.0 International license.

**FIG 3:**
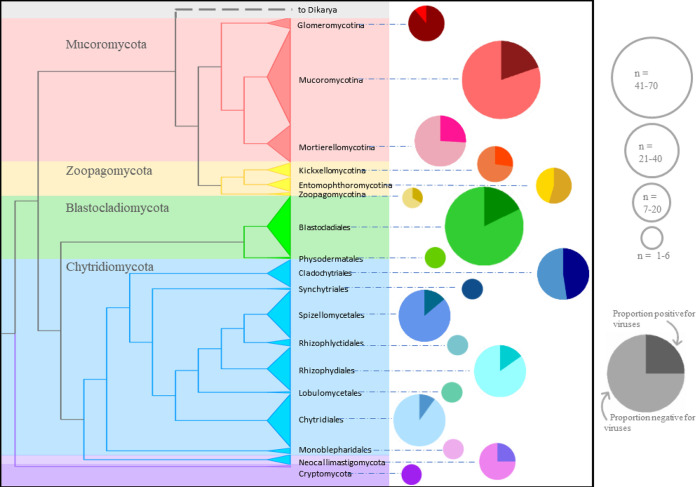
Cladogram of the organisms screened for viruses by both methods in this study. The size of the collapsed clades is proportional to the number of isolates screened. Pie charts indicate the proportions of isolates in each taxon that were viral positive (darker shade); pie charts are sized according to number of isolates screened. Whole pies had a 0% infection rate.

All sampled phyla were hosts to viruses except the poorly sampled Cryptomycota. Infection prevalence at the phylum level ranged from 15.9 to 40.0% and 0 to 89% at the subphylum and order levels but was highest in Glomeromycotina, Entomophthoromycotina, and Cladochytriales (Table 1 and Fig. 3). Glomeromycotina are also notorious hosts of endosymbiotic bacteria which may have originated by the invasion of free-living bacteria following hyphal damage by herbivores or other fungi (21); it is possible that mycoviruses similarly originated in these fungi by invasion through damaged cell walls. In the zoosporic fungi, we predicted that polycentric organisms would be disproportionately virally infected since this mode should support viral transmission more frequently than monocentric growth. In monocentric development a zoospore encysts on a substrate and develops into a single zoosporangium—the structure which will ultimately cleave into many zoospores. Polycentric development, on the other hand, involves a single zoospore producing multiple zoosporangia with cytoplasmic continuity via aseptate rhizomycelium. Thus, a single viral infection in a polycentric organism could lead to many spores being infected, while monocentric development may allow for clearing of viral infection through selection between zoospores. Within the Chytridiomycota, the percentage of polycentric organisms that were viral positive (46.2%, *n* = 26) was significantly greater (*P* < 0.00004, Fisher exact test) than monocentric organisms (9.2%, *n* = 120). Ten of the twelve positive polycentric isolates were from the order Cladochytriales, however, and consequently we were unable to determine whether this trend is a result of the morphological trait or susceptibility traits that track the host phylogeny without additional data from non-Cladochytrialean polycentric organisms. Although the sample size is inadequate for definitive results regarding Neocallimastigomycota, viral infection did not favor polycentric over monocentric organisms in this phylum (polycentric *n* = 4 and monocentric *n* = 4, 25% of each was viral positive).

**TABLE 1:** Percent viral prevalence based on combined *in vitro* and *in silico* screening, by phyla and subphyla, or order

Taxonomic group	Sample size (*n*)	Virus prevalence (%)
**Mucoromycota**	107	27.1
Glomeromycotina	9	88.9
Mucoromycotina	71	19.7
Mortierellomycotina	27	25.9
**Zoopagomycota**	25	40.0
Entomophthoromycotina	11	54.5
Kickxellomycotina	11	27.3
Zoopagomycotina	3	33.3
**Blastocladiomycota**	44	15.9
Blastocladiales	42	16.7
Physodermatales	2	0
**Chytridiomycota**	146	16.4
Monoblepharidales	5	0
Cladochytriales	21	47.6
Synchytriales	2	0
Chytridiales	40	10.0
Lobulomycetales	2	0
Rhizophydiales	35	14.3
Rhizophlyctidales	6	0
Spizellomycetales	36	13.9
**Neocallimastigomycota**	8	25.0
**Cryptomycota**	2	0
Total	333	21.6

Early in the study of mycoviruses, Bozarth (22) estimated 10 to 15% infection prevalence for fungal cultures predominantly made up of dikaryotic lineages but including some organisms in the Mucoromycota and Blastocladiomycota. A recent *in silico* survey of mycoviruses in Pezizomycotina (Ascomycota) revealed infection prevalence ranging up to 50% in some classes but an overall rate of about 8% (47/569) for the subphylum (17). Our estimate is approximately 22% (Table 1) across the early-diverging phyla. Interestingly, we also found substantial variation across taxa (Table 1 and Fig. 3). Among the phyla with the highest infection rates, Mucoromycota and Zoopagomycota, viral prevalence at the subphylum level ranged from 19.7 to 88.9% (Table 1), a surprising difference from the 8% prevalence found for the same taxonomic level, Pezizomycotina, in Ascomycota. These data sets were not explicitly controlled for geographic origin of isolates, which could influence prevalence if, for example, some geographic regions had a higher proportion of viruses relative to others. However, the isolates in our study predominantly originate from distinct populations across the globe (see Table S1 in the supplemental material). Our results indicate higher prevalence in some subphyla of basal fungal lineages compared to Pezizomycotina. From our comparison of viral prevalence at the subphylum level between Pezizomycotina (Ascomycota) versus the early-diverging lineages, it is tempting to speculate on differing viral prevalence at the phylum level. However, a taxonomically thorough and geographically controlled study of viral prevalence in the other subphyla of Ascomycota and in Basidiomycota is needed in order to make a direct comparison.

In addition, anecdotal evidence suggests mycoviruses are commonly lost through culturing. Our estimates from the *in vitro* assays are thus perhaps underestimates of the true infection load in nature. Nonetheless, it is compelling to consider the implications of years or decades of stable maintenance of mycoviruses in culture on how the viruses affect the fungi. Future studies are needed to better characterize the effects of these mycoviruses on these phylogenetically diverse hosts.

Taken together, these findings indicate a strong phylogenetic component to viral prevalence. The earlier-diverging fungal lineages surveyed in this study typically share the trait of coenocytic mycelia, which may benefit the transmission of obligate symbionts and thus facilitate mycoviral infection; if true, it is possible that septa evolved, at least in part, as a viral defense mechanism to limit the spread of viruses throughout a mycelium.

### Comparison of screening approaches.

The results of screening the same isolate by chromatography and transcriptome-mining were mostly in agreement (i.e., at least one band of dsRNA was present by chromatography and RdRp sequence(s) were identified *in silico* or no dsRNA was present by chromatography, and no RdRp sequences were identified) (85.7%, *n* = 21). Of the three isolates where the two methods varied in detection, two were found positive *in vitro* but negative *in silico*, and one with the inverse result. For *Mortierella humilis* (PMI 1414), where the *in silico* method revealed viral sequences but the *in vitro* method did not, RT-PCR confirmed viral presence in our culture. From the transcriptome data, we determined the virally derived sequences made up 2.58 transcripts per million. If we consider this a proxy of viral titer, the low viral abundance in the host likely accounts for the initial negative result obtained with the *in vitro* method. From *Umbelopsis nana* TLT204, which was positive by in-house chromatography but negative *in silico*, we sequenced purified dsRNA and assembled a toti-like viral contig containing a complete RdRp domain and an L-A virus major coat-protein domain, confirming a viral presence in our culture. Most likely, the virus was lost through subculture of this isolate in the laboratory in which transcriptome analysis was performed.

Paired comparison of screening approaches suggests minimal discrepancies, but more viruses overall were detected by the *in silico* method (33.3% positive) than *in vitro* (17% positive). The *in silico* method is likely to be more sensitive than the *in vitro* method, but it is possible that the higher virus detection rate *in silico* was a result of phylogenetic skews of the data sets rather than methodology. Approximately 73% of the cultures screened *in vitro* were in the Chytridiomycota or Blastocladiomycota, the lesser-infected phyla. Accordingly, 64% of the isolates screened *in silico* were in the Mucoromycota or Zoopagomycota, the greater-infected phyla. Thus, we recommend screening by transcriptome-mining when the data are available, but the chromatography approach is a good alternative for very cost-effective initial screening.

### Mycoviral diversity.

**FIG 4:**
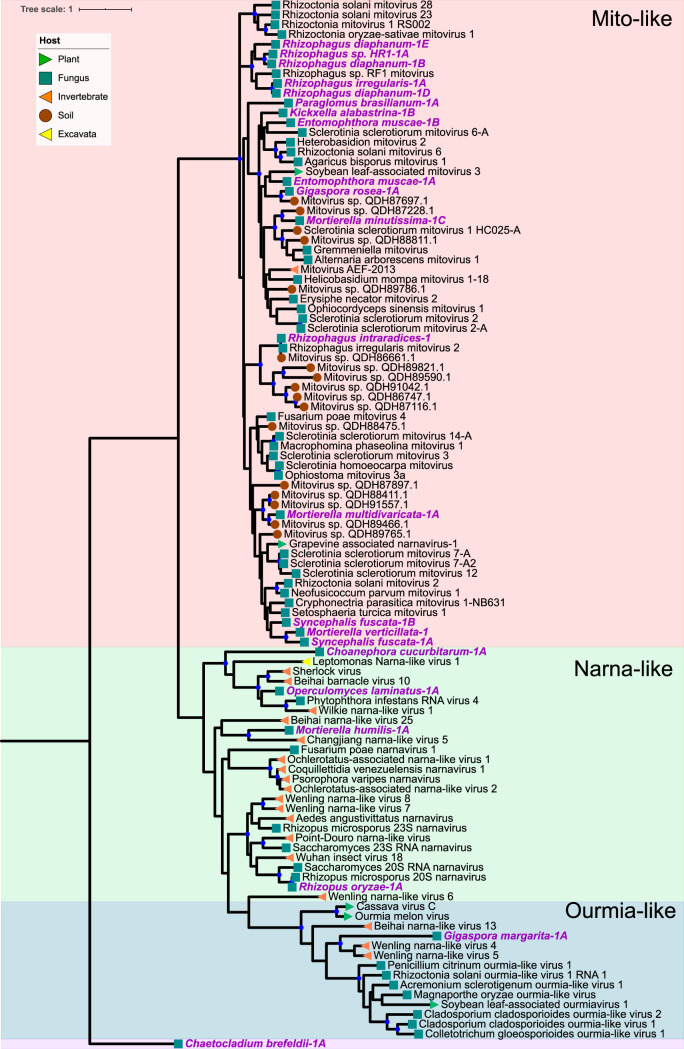
Maximum-likelihood tree of new mycovirus RdRps with top blast hits (included in tree) to viruses in “branch 1” of Riboviria. The best model of amino acid substitution for this model was determined to be LG+G according to Prottest v3.4. Host taxonomy is indicated by branch symbols, and viral taxonomic groupings are indicated by a shaded background. Solid blue circles indicate well-supported nodes with ≥70% bootstrap support. New sequences are indicated by purple text.

**FIG 5:**
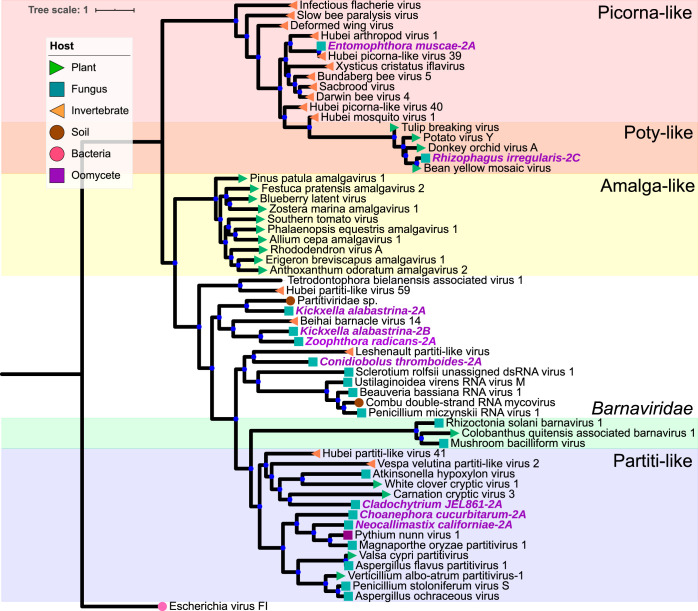
Maximum-likelihood tree of new mycovirus RdRps with top blast hits (included in tree) to viruses in “branch 2” of Riboviria. The best model of amino acid substitution for this model was determined to be VT+I+G according to Prottest v3.4. Host taxonomy is indicated by branch symbols, and viral taxonomic groupings are indicated by a shaded background. Solid blue circles indicate well-supported nodes with ≥70% bootstrap support. New sequences are indicated by purple tip labels.

**FIG 6:**
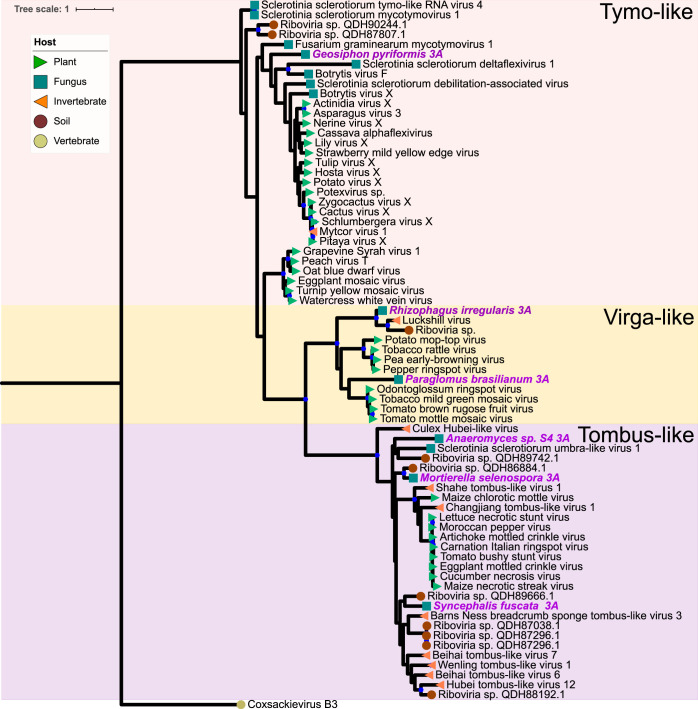
Maximum-likelihood tree of new mycovirus RdRps with top blast hits (included in tree) to viruses in “branch 3” of Riboviria. The best model of amino acid substitution for this model was determined to be LG+G according to Prottest v3.4. Host taxonomy is indicated by branch symbols, and viral taxonomonic groupings are indicated by a shaded background. Solid blue circles indicate well-supported nodes with ≥70% bootstrap support. New sequences are indicated by purple tip labels.

**FIG 7:**
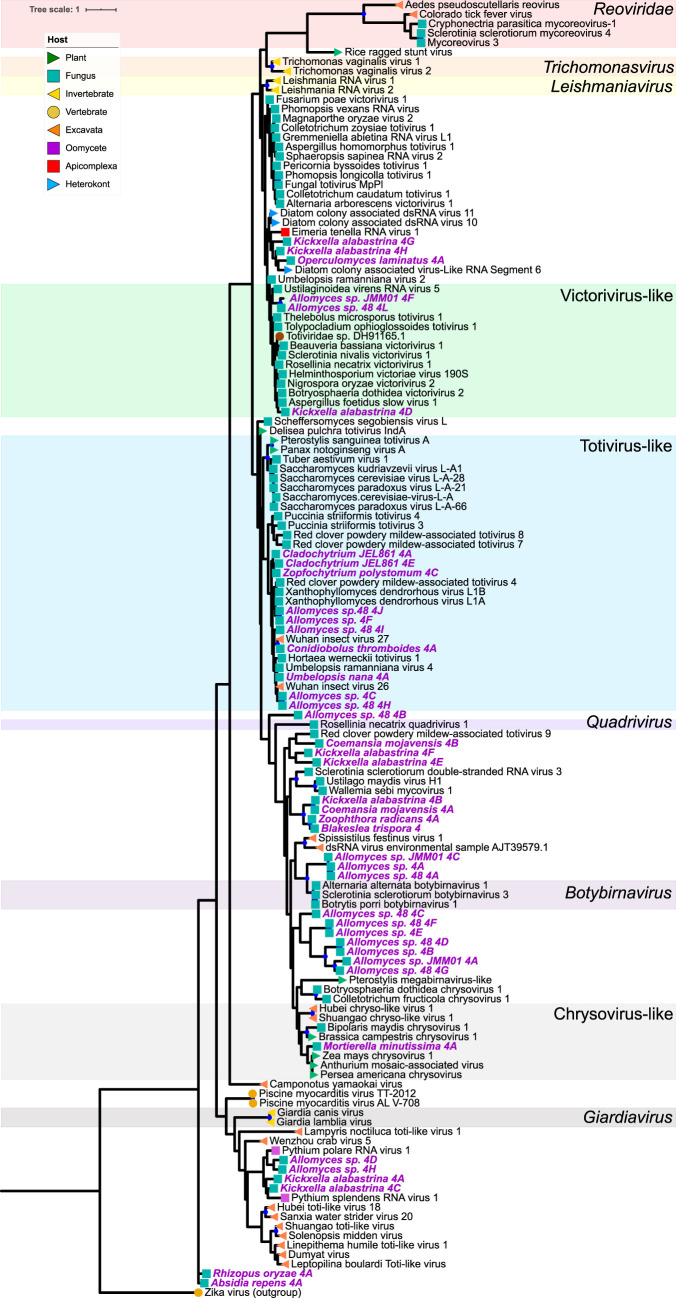
Maximum-likelihood tree of new mycovirus RdRps with top blast hits (included in tree) to viruses in “branch 4” of Riboviria. The best model of amino acid substitution for this model was determined to be LG+G according to Prottest v3.4. Host taxonomy is indicated by branch symbols, and viral taxonomic groupings are indicated by a shaded background. Solid blue circles indicate well-supported nodes with ≥70% bootstrap support. New sequences are indicated by purple tip labels.

**FIG 8:**
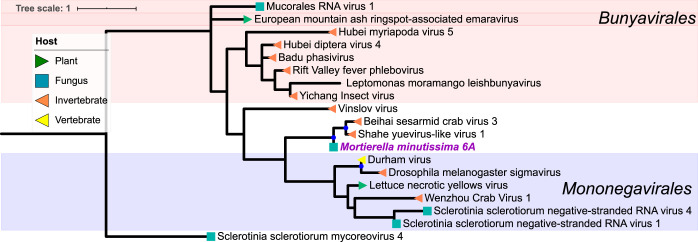
Maximum-likelihood tree of a new mycovirus RdRp with top blast hits (included in tree) to viruses in “branch 5” of Riboviria. The best model of amino acid substitution for this model was determined to be VT+G according to Prottest v3.4. Host taxonomy is indicated by branch symbols, and viral taxonomic groupings are indicated by a shaded background. Solid blue circles indicate well-supported nodes with ≥70% bootstrap support. New sequences are indicated by purple tip labels.

**FIG 9:**
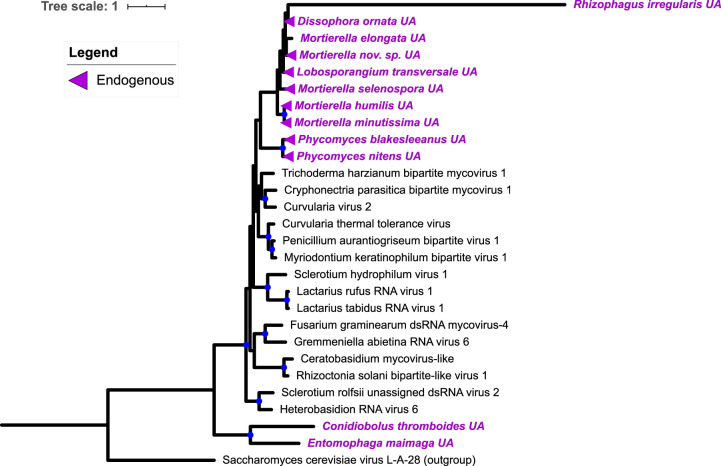
Maximum-likelihood tree of new mycovirus RdRps with top blast hits (included in tree) unassigned by current viral taxonomy. The best model of amino acid substitution for this model was determined to be LG +G according to Prottest v3.4. All viruses have fungal hosts. New sequences are indicated by purple tip labels. Blue circles indicate nodes with ≥70% bootstrap support. Triangles indicate novel virus-like sequences determined to be endogenous in the host genome. A DNA-based genome for *Mortierella elongata* has yet to be sequenced, and so we cannot conclude that *Mortierella elongata UA virus* is endogenous, although it appears likely.

We found mycoviruses in all five “branches” of the Riboviria (RNA virus) tree published by Wolf et al. (23) (Fig. 4 to 8). Many of these newly found viruses will need to be described as new taxa at the levels of genus, family, and order. In addition, some viruses, including a group of related, endogenous virus-like sequences, represent novel diversity such that they were unable to be assigned to a branch, though they show sequence similarity to other unplaced mycoviruses (Fig. 9). Coinfection of a single host by multiple viruses was very common (mean and median number of viruses per host = 2.4 and 1.5, respectively). Even after filtering by our conservative criteria, a single host, *Kickxella alabastrina* (Kickxellomycotina), was found to harbor at least 11 unique viruses. Such a result would be unsurprising in macrobes, such as humans but is staggering for a microfungus.

**(i) Riboviria: “branch 1.”** Branch 1 (Fig. 4) is composed of positive-sense ssRNA viruses, including the bacterium-infecting *Leviviridae*, the mitochondrion-infecting mitoviruses (*Narnaviridae*), and the cytoplasmic narnaviruses (*Narnaviridae*) and ourmiaviruses (*Botourmiaviridae*). In total, 22 viruses were assigned to branch 1 based on blast similarity. A complete RdRp gene from the transcriptome of *Chaetocladium brefeldii* was most similar to viruses in family *Leviviridae*. These viruses are currently only known from bacteria, so it is possible, and perhaps likely, that this contig derives from the viruses of a bacterial endosymbiont of *C. brefeldii*.

Interestingly, the new viruses in this group are mostly hosted by fungi in the Mucoromycota or Zoopagomycota. Previous evidence suggests the origin of mitoviruses was a common ancestor of Mucoromyota and Zoopagomycota, which was followed by cospeciation of host and mitoviruses and horizontal transmission to plants (13, 24). Our findings support this hypothesis, but sequence data are still limited for viruses in Chytridiomycota, Blastocladiomycota, and Neocallimastigomycota. Additional sequencing of the viral positives in those groups found by chromatography in this study will more strongly support or refute this hypothesis.

**(ii) Riboviria: “branch 2.”** The picornavirus supergroup makes up branch 2 (Fig. 5), which also includes *Partitiviridae*, *Amalgaviridae*, *Barnaviridae*, and *Potyviridae*. In all, nine viruses were assigned to branch 2 based on BLAST similarity. The hosts of these contigs represent the Mucoromycota, Zoopagomycota, Neocallimastigomycota, and Chytridiomycota. A virus of *Entomophthora muscae* nested within the picorna-like viruses, specifically in the genus *Iflavirus*, which is only known to infect insects. Thus, it may have originated by cross-kingdom horizontal transfer. A virus of the arbuscular mycorrhizal fungus *Rhizophagus irregularis* (Glomeromycotina) is nested within the plant-infecting *Potyviruses* and likely also arose by horizontal transmission given the tight mutualism and nutrient exchange between *R. irregularis* and its plant hosts.

**(iii) Riboviria: “branch 3.”** Branch 3 (Fig. 6) includes the alphavirus and flavivirus supergroups. In total, six contigs were assigned to this branch based on BLAST similarity. We found three new tombusvirus-like viruses, hosted by *Mortierella selenospora* (Mucoromycota), *Syncephalis fuscata* (Zoopagomycota), and *Anaeromyces* sp. (Neocallimastigomycota). Two arbuscular mycorrhizal fungi, *R. irregularis* and *Paraglomus brasilianum*, contained viruses related to the predominantly plant-hosted viruses in *Virgaviridae*. Another Glomeromycotina fungus, *Geosiphon pyriformis*, was host to a tymo-like virus positioned basally in the order *Tymovirales* and may represent a new family in this order.

**(iv) Riboviria: “branch 4.”** The largest number of new contigs were assigned to branch 4 (Fig. 7), which includes *Totiviridae*, *Chrysoviridae*, *Quadriviridae*, and *Reoviridae*. In total, our analysis assigned 41 contigs to this group. Hosts are largely represented by Chytridiomycota and Blastocladiomycota, including substantial coinfection within the same individual, but also by Mucoromycota and Zoopagomycota.

Ten of these viruses group within the genus *Totivirus*, and three new viruses grouped within the genus *Victorivirus*, both genera belonging to the family *Totiviridae*. By our analysis, these two genera are composed of fungal viruses truly spanning the Kingdom, including hosts in Chytridiomycota, Blastocladiomycota, Zoopagomycota, Mucoromycota, Ascomycota, and Basidiomycota. This finding may align with the cospeciation hypothesis for this viral family: Göker et al. presented evidence that *Totiviridae* speciate through codivergence with their hosts (25). The predominance of totiviridae-like sequences in early-diverging lineages supports this hypothesis and suggests that an early fungal ancestor harbored ancestors of *Totiviridae.* However, this origin hypothesis does not preclude occasional horizontal transfer, many instances of which are suggested by our phylogenetic tree. Further, this viral family also include two genera, *Leishmaniavirus* and *Giardiavirus*, that infect protozoa; since protozoans and Fungi are polyphyletic, ancient horizontal transfer between host groups is likely.

Basal to *Victorivirus*, a clade formed including viruses from *K. alabastrina*, *Operculomyces laminatus*, the ampicomplexan *Eimeria tenella*, and multiple-diatom colonies. This finding aligned with our prediction of close-relatedness between formerly reported diatom-associated viruses and the newly reported sequences from Chytridiomycota, a group that contains diatom parasites, but lacked bootstrap support. Viruses from multiple isolates of *Allomyces* sp. form a clade sister to *Botybirnavirus*, likely a new genus. Generally, many of the new sequences in this branch represent novel diversity that will require deeper viral characterization to phylogenetically classify with confidence.

**(v) Riboviria: “branch 5.”** We identified just one viral contig in branch 5, which includes the negative-sense ssRNA viruses *Mononegavirales* and *Bunyavirales* (Fig. 8). The virus found in *Mortierella minutissima* is most closely related to the undersampled yueviruses known from insect hosts. Viruses with fungal hosts have not previously been reported from this, or related, lineages and the *M. minutissima* virus may represent an undescribed family.

**(vi) Riboviria: unassigned.** Some viral contigs either did not have significant BLAST hits or hit to known viruses currently unassigned to a taxonomic grouping (*n* = 12) (Fig. 9). These new viral contigs appear to be related to unplaced “bipartite mycoviruses” and Curvularia thermotolance virus, known to form a tripartite mutualism with its fungal host and a plant (26). Among these viruses we uncovered is a novel lineage found endogenous in hosts’ genomes. Intriguingly, we identified a highly supported clade of virus-like sequences only in the genomes of fungi in the Mucoromycota, including two species of *Phycomyces*, *Dissophora ornata*, *Lobosporangium transversale*, and multiple *Mortierella* spp. (Fig. 9). Conserved RdRp domains were identifiable in all nine of these sequences; all lacked specific hits to reverse transcriptome domains and thus are not believed to be retroviruses. Rather, the phylogenetic conservation of these endogenous virus-like sequences suggests that, before the divergence of Mucorales and Mortierellales, an endogenization event, in which a viral gene was reverse transcribed and integrated into the host genome, occurred that was conserved in members of Phycomycetaceae and Mortierellales. Integration of viral genes into host genomes is a well-known phenomenon generally, and the transfer of genes from dsRNA viruses to Fungi has been reported by others (27–29). We identified the new sequences via their mRNA transcripts with our *in silico* approach, which confirms their expression. Thus, this previously unreported instance of viral endogenization seemingly resulted in a novel fungal gene, the function of which remains to be tested.

### Conclusions.

The results of this study provide a new perspective on mycoviral prevalence in Fungi and the phylogenetic diversity of viruses. The results also aid in the identification of hosts of environmentally derived virus samples. For example, many of our mitovirus-like viral contigs appear to be related to *Mitovirus* species from soils; we suggest that these soil-derived mitoviruses are likely hosted by fungi in Mucoromycota.

The ecological implications of viruses in these deep branches of the fungal tree are currently only a matter of speculation, but their role in natural ecosystems may be of great importance. The host organisms studied embody a broad diversity of ecological niches including saprotrophs, plant mutualists, obligate and opportunistic pathogens, and parasites of plants, invertebrates, animals, protists, and other fungi. Even slight effects on the growth rate of saprotrophs, for instance, could have significant impact on nutrient cycling on a global scale.

By searching unexplored and underexplored fungal lineages, we uncovered novel mycoviral diversity and discovered that fungal viruses are indeed ubiquitous throughout the fungal kingdom, detected now in nearly every phylum. Our data suggest that early-diverging lineages may harbor greater viral prevalence than the Dikarya, but there is wide variation across lineages. A caveat of comparisons across major taxonomic groups is that without broad and deliberate taxonomic and geographic sampling, comparison of rates of infection are subject to high error in estimation. Further, by searching publicly available transcriptomes, as well as cultures from collections that are distributed to researchers globally, we learned that mycoviruses are abundant in research organisms used in laboratories worldwide. Mycoviruses are known not only to be persistent and often asymptomatic but also to cause variable phenotypic alterations such as in pigmentation, growth rate, and virulence. The implications for cryptic mycoviral infection in laboratory cultures are currently unknown but provide a guide for future studies into mycovirus origins and ecological functions.

## MATERIALS AND METHODS

### *In vitro* screening. (i) Fungal cultures.

Cultures were obtained from the Collection of Zoosporic Eufungi at the University of Michigan (CZEUM; recently founded from the Joyce Longcore University of Maine Collection [JEL] and the University of Alabama Chytrid Culture Collection [UACCC]), the Agricultural Research Service Culture Collection (NRRL), the Collection of Entomopathogenic Fungal Cultures (ARSEF), and the collections of the authors (see Table S1 in the supplemental material). We grew isolates in media appropriate for their nutritional needs until sufficient biomass accumulated (3 days to 2 weeks, depending on the species), harvested tissue, and ground it by sterile mortar and pestle in liquid nitrogen. With every batch of fungi screened, we harvested and processed a mycovirus-infected strain of Ustilago maydis as a positive control for degradation of mycoviruses by RNases.

### (ii) Column preparation and dsRNA extraction.

We screened cultures for RNA mycoviruses by dsRNA extraction and purification by cellulose chromatography as described by Okada et al. (30) with slight modifications. Before RNA extraction, we prepared columns by piercing the bottom of a 0.5-ml tube with an 18-gauge needle, packing it with ∼90 mg of cellulose D powder (Advantec), placing it in a 2-ml microcentrifuge tube, and adding 400 μl of freshly prepared 1× sodium chloride-Tris-EDTA (STE) with 16% ethanol. Immediately before use, we centrifuged the columns briefly and discarded the flowthrough. We extracted RNA by adding one ml of TRIzol reagent (Invitrogen) to ∼0.5 mg finely ground frozen tissue and either freezing at –20°C for later processing or incubating for 10 min at room temperature, adding 200 μl of chloroform, followed by mixing by inversion, incubation for 3 min at room temperature, and centrifugation at 12,000 × *g* at 4°C for 15 min. We made a 16% ethanol solution with the supernatant, added it to the cellulose column, collected and discarded the flowthrough, washed the column three times with 400 μl of 1× STE with 16% ethanol, thoroughly dried it by centrifugation, and eluted the columns with 400 μl of 1× STE. We precipitated dsRNA by adding 40 μl of 3 M sodium acetate and 1 ml of ethanol, centrifugation at 15,000 × *g* for 5 min, pipetting off the supernatant, and allowing the tubes to dry before reconstitution with water. We treated samples with S1 nuclease and DNase 1 according to the manufacturer’s instruction before visualization using agarose gel electrophoresis. We considered samples positive if a band was visible (Fig. 2).

### *In silico* screening.

We obtained unassembled RNA-Seq data from the SRA database (see Table S2 in the supplemental material), stringently filtered raw reads for quality (minimum [min] phred score 20) using the fastxtoolkit (31), and assembled contigs *de novo* with Trinity assembler (32). We predicted open reading frames (ORFs) with Transdecoder (33) using default parameters and queried the protein translations with hmmscan (34) against a custom RdRp HMM database. We constructed the RdRp database as in Gilbert 2019: we downloaded entire alignments of Pfam families RdRp_1, RdRp_2, RdRp_3, RdRp_4, and Mito_RdRp and generated HMM profiles from each using hmmbuild (HMMER2; hmmer.org). We further queried each RdRp profile hit found in the transcriptome ORFs with TBLASTN and BLASTP to the NCBI nt and nr databases (downloaded 18 September 2019), respectively, and considered isolates positive for viral infection if the resultant hits were viral sequences with an E value < e^−10^. To ensure the virus was exogenous to the host genome, we blasted viral contigs against the hosts’ genome when available or, if this was unavailable, the genome of the closest relative.

10.1128/mBio.02027-20.2 TABLE S2 Transcriptomes retrieved from SRA database. Download Table S2, DOCX file, 0.02 MB. Copyright © 2020 Myers et al. 2020 Myers et al. This content is distributed under the terms of the Creative Commons Attribution 4.0 International license.

To improve assemblies, we used the nucleotide sequence of the Trinity-assembled viral contig as the seed for contig extension with PRICE (35) with the parameters of a minimum 30-nucleotide overlap for mini-assembly, a minimum 80% identity for contig-edge assembly, a 90% identity to starting contigs, and for 10 cycles, using loosely filtered raw reads (min phred score 5) (36). This most often resulted in contigs that were unchanged after 10 cycles, which we considered complete. If contigs were updated in the procedure, we ran 10 additional cycles with the updated contig as the starting seed.

As a final quality control check of our virus genomes, we reassembled the viromes of a subset of isolates by first aligning loosely filtered raw reads (min phred score 5) to a reference genome using STAR (37), assembled unmapped reads *de novo* using Trinity and then continued the pipeline exactly as described above. In all cases, at least one viral contig was extended by this method, but the RdRp region was most often unchanged. For additional informatics details and code, see https://github.com/jimyers/Mycoviruses-in-early-diverging-fungal-lineages.

### Mycovirus sequencing. (i) Pacific Biosciences sequencing.

We prepared purified dsRNAs obtained from *in vitro* screens of *Allomyces* sp. strain JMM01, *Allomyces* sp. strain DJ-02, *Allomyces* sp. strain DJ-07, Zopfochytrium polystomum WB228, and Ustilago maydis (as a control) as described by M. J. Roossinck in 2010 (43) with slight modifications. Briefly, we purified dsRNAs by cellulose chromatography as described above, except the final elution was performed with 20 μl of 2× STE (pH 8.0). We mixed 1 μl of dsRNA with 7 μl of H_2_O and 2 μl of tagged random 12-mer at 20 μM (5′-ACCTTCGGATCCTCC-N12-3′), placed the tube in boiling water for 2 min to melt strands, immediately quenched the sample on ice, used the Omniscript reverse transcription kit according to the manufacturer’s protocol, incubated the tubes on ice for 10 min followed by 60 min at 50°C, removed the unreacted template with 2 μl of RNase A (5 mg/ml), and then incubated the sample at room temperature for 15 min, followed 3 min at 80°C to denature the enzymes. We used a Qiagen PCR purification kit according to the manufacturer’s protocols to clean up PCR products and amplified them by PCR with individually barcoded primers using GoTaq polymerase, followed by gel extraction using a Qiagen gel extraction kit and the manufacturer’s protocol. For each isolate, we sequenced 120 ng of product on a PacBio RSII at the University of Michigan Advanced Genomics Core (UM-AGC). We analyzed reads with SMRT Portal (parameters: minimum barcode score 22, minimum of 5 passes, minimum 90% accuracy) and removed adapters with cutadapt, followed by read correction, trimming, and assembly with Canu (v1.3) (parameters: minOverlapLength = 100, minReadLength = 150, errorRate = 0.035, estimated genome size of 10,000 bp). For quality control, we compared a *U. maydis* contig to the GenBank *U. maydis* H1 cap-pol fusion nucleotide sequence (NC_003823; fungal isolate unknown) which matched with a 99% query cover and a 93% identity.

### (ii) (ds)RNA-Seq and total RNA-Seq.

We submitted purified dsRNAs from *in vitro* screens of *Cladochytrium* sp. JEL861, *Allomyces arbusculus* North Carolina 2, *Umbelopsis nana* TLT 204, and *U. maydis* (as a control) to the UM-AGC for 2 × 150 sequencing on Illumina MiSeq with the according to modifications to the manufacturer’s instructions for library preparation as described by Sasai et al. (38): fragmentation was conducted with 87.5 ng of purified dsRNA for 20 min at 94°C in first-strand synthesis buffer with random primers, and the first-strand synthesis reaction was conducted for 30 min at 42°C (38). We submitted total RNA extractions of *Allomyces* sp. JMM01 and *Z. polystomum* WB228 to the UM-AGC for library preparation using the Illumina TruSeq stranded mRNA protocol and 2 × 50 sequencing using an Illumina HiSeq 4000. All RNA-Seq was processed to remove adapters and low-quality sequences from paired-end data using trimgalore (min quality threshold 5) (https://github.com/FelixKrueger/TrimGalore), followed by assembly *de novo* with Trinity. We performed ORF prediction, HMM queries for RdRp homologs, and PRICE extension as described above.

For transcriptomes sequenced by JGI and not published previously (see Table S2), stranded cDNA libraries were generated using the Illumina Truseq stranded RNA LT kit. mRNA was purified from 1 μg of total RNA using magnetic beads containing poly(T) oligonucleotides, fragmented, and reverse transcribed using random hexamers and SSII (Invitrogen), followed by second-strand synthesis. The fragmented cDNA was treated with end-pair, A-tailing, adapter ligation, and 8 to 10 cycles of PCR. The prepared library was quantified using KAPA Biosystem’s next-generation sequencing library qPCR kit and run on a Roche LightCycler 480 real-time PCR instrument, multiplexed with other libraries, and the pool of libraries was then sequenced on an Illumina platform (HiSeq 2000/2500 or NovaSeq) following a 2 × 150 indexed run recipe.

### Comparison of screening approaches.

One isolate, *Mortierella humilis* PMI 1414, produced negative results by *in vitro* virus screen but positive results by the *in silico* method. We designed virus-specific primers based on the *in silico* results and conducted RT-PCR using SuperScript IV reverse transcriptase (Thermo Fisher) according to the manufacturer’s protocol. For a proxy of viral titer, we calculated the abundance of viral transcripts generated in the transcriptome with the align_and_estimate_abundance.pl script of the Trinity package (https://github.com/trinityrnaseq/trinityrnaseq), employing bowtie for alignment and the RSEM estimation method.

### Mycovirus sequence analysis and phylogenetics.

We classified each new mycoviral sequence by top blast hit and grouped them into clusters corresponding to “branches” described in the most recent RNA virus phylogeny (23). ORFs, including each RdRp gene, were predicted and translated using the universal genetic code except for sequences with top blast hits to mitoviruses, for which we used the standard mitochondrial genetic code.

Despite our best attempts to resolve assemblies, some remained fragmented. To avoid overreporting, we applied conservative criteria to limit the number of viruses reported for each isolate in a biologically relevant manner. The criteria for inclusion in our analyses were as follows: if only one viral contig was identified per strain per branch, we included it. For branches with more than one viral contig per isolate, all contigs with >60% coverage of the RdRp conserved domain were included, or if all contigs for that branch contain <60% of the RdRp, then we kept the contig with highest coverage. Three isolates had only two viral contigs assembled, one of which contained the C terminus of the RdRp and the other contained the N terminus (*B. trispora*, *M. verticillata*, and *R. intraradices*). In these three instances, we concatenated the two contigs.

For each branch, we inferred RdRp gene trees with viral contigs that met our criteria, their top BLAST hits, and reference sequences (Table S3). A sixth tree (Fig. 9) includes sequences with BLAST similarities to viruses currently unassigned by viral taxonomy. We aligned sequences with MAFFT version 7 using the E-INS-i algorithm (39) and trimmed the resulting alignments using the -automated1 method in TrimAl (40). We determined the best-fit model of amino acid substitution for each alignment with ProtTest 3.4 (41) and reconstructed trees with the maximum-likelihood approach implemented in RaxML by the rapid bootstrap analysis (-f a) with 100 replicates (42).

10.1128/mBio.02027-20.3 TABLE S3 GenBank accessions of reference viral sequences. Download Table S3, XLSX file, 0.03 MB. Copyright © 2020 Myers et al. 2020 Myers et al. This content is distributed under the terms of the Creative Commons Attribution 4.0 International license.

### Data availability.

The sequences generated for this study can be found in GenBank BioProject PRJNA657856. Sequence alignments, HMMs, and code can be found on github at https://github.com/jimyers/Mycoviruses-in-early-diverging-fungal-lineages.

## References

[1] Ghabrial SA, Castón JR, Jiang D, Nibert ML, Suzuki N 2015 50-plus years of fungal viruses. Virology 479-480:356–368. doi:10.1016/j.virol.2015.02.034. 25771805

[2] Son M, Yu J, Kim KH 2015 Five questions about mycoviruses. PLoS Pathog 11:e1005172. doi:10.1371/journal.ppat.1005172. 26539725 PMC4635019

[3] James TY, Stajich JE, Hittinger CT, Rokas A 2020 Toward a fully resolved fungal tree of life. Annu Rev Microbiol 74:291–313. doi:10.1146/annurev-micro-022020-051835. 32660385

[4] Spatafora JW, Chang Y, Benny GL, Lazarus K, Smith ME, Berbee ML, Bonito G, Corradi N, Grigoriev I, Gryganskyi A, James TY, O’Donnell K, Roberson RW, Taylor TN, Uehling J, Vilgalys R, White MM, Stajich JE 2016 A phylum-level phylogenetic classification of zygomycete fungi based on genome-scale data. Mycologia 108:1028–1046. doi:10.3852/16-042. 27738200 PMC6078412

[5] Hibbett DS, Binder M, Bischoff JF, Blackwell M, Cannon PF, Eriksson OE, Huhndorf S, James T, Kirk PM, Lücking R, Thorsten Lumbsch H, Lutzoni F, Matheny PB, McLaughlin DJ, Powell MJ, Redhead S, Schoch CL, Spatafora JW, Stalpers JA, Vilgalys R, Aime MC, Aptroot A, Bauer R, 2007 A higher-level phylogenetic classification of the Fungi. Mycol Res 111:509–547. doi:10.1016/j.mycres.2007.03.004. 17572334

[6] Khandjian EW, Roos U, Timberlake WE, Eder L, Turian G 1974 RNA virus-like particles in the chytriomycete *Allomyces arbuscula*. Arch Microbiol 101:351–356. doi:10.1007/BF00455951. 4477001

[7] Papp T, Nyilasi I, Fekete C, Ferenczy L, Vágvölgyi C 2001 Presence of double-stranded RNA and virus-like particles in *Rhizopus* isolates. Can J Microbiol 47:443–447. doi:10.1139/w01-020. 11400735

[8] Espino-Vázquez AN, Bermúdez-Barrientos JR, Cabrera-Rangel JF, Córdova-López G, Cardoso-Martínez F, Martínez-Vázquez A, Camarena-Pozos DA, Mondo SJ, Pawlowska TE, Abreu-Goodger C, Partida-Martínez LP 2020 Narnaviruses: novel players in fungal–bacterial symbioses. ISME J 14:1743–1754. doi:10.1038/s41396-020-0638-y. 32269378 PMC7305303

[9] Kitahara R, Ikeda Y, Shimura H, Masuta C, Ezawa T 2014 A unique mitovirus from Glomeromycota, the phylum of arbuscular mycorrhizal fungi. Arch Virol 159:2157–2160. doi:10.1007/s00705-014-1999-1. 24532299

[10] Neupane A, Feng C, Feng J, Kafle A, Bücking H, Lee Marzano S-Y 2018 Metatranscriptomic analysis and in silico approach identified mycoviruses in the arbuscular mycorrhizal fungus rhizophagus spp. Viruses 10:707. doi:10.3390/v10120707. PMC6316171 30545059

[11] Kartali T, Nyilasi I, Szabó B, Kocsubé S, Patai R, Polgár TF, Nagy G, Vágvölgyi C, Papp T 2019 Detection and molecular characterization of novel dsRNA viruses related to the *Totiviridae* family in *Umbelopsis ramanniana*. Front Cell Infect Microbiol 9:249–213. doi:10.3389/fcimb.2019.00249. 31380294 PMC6644447

[12] Coyle MC, Elya CN, Bronski M, Eisen MB 2018 Entomophthovirus: an insect-derived iflavirus that infects a behavior manipulating fungal pathogen of dipterans. bioRxiv 371526. doi:10.1101/371526. PMC11457076 39158097

[13] Nibert ML, Debat HJ, Manny AR, Grigoriev IV, De Fine Licht HH 2019 Mitovirus and mitochondrial coding sequences from basal fungus *Entomophthora muscae*. Viruses 11:351–358. doi:10.3390/v11040351. PMC6520771 30999558

[14] Morris TJ, Dodds JA 1979 Isolation and analysis of double-stranded RNA from virus-infected plant and fungal tissue. Phytopathology 69:854–858. 10.1094/Phyto-69-854 40934407

[15] Nerva L, Ciuffo M, Vallino M, Margaria P, Varese GC, Gnavi G, Turina M 2016 Multiple approaches for the detection and characterization of viral and plasmid symbionts from a collection of marine fungi. Virus Res 219:22–38. doi:10.1016/j.virusres.2015.10.028. 26546154

[16] Marzano SYL, Domier LL 2016 Reprint of “Novel mycoviruses discovered from metatranscriptomics survey of soybean phyllosphere phytobiomes.” Virus Res 219:11–21. doi:10.1016/j.virusres.2016.05.012. 27208848

[17] Gilbert KB, Holcomb EE, Allscheid RL, Carrington JC 2019 Hiding in plain sight: new virus genomes discovered via a systematic analysis of fungal public transcriptomes. PLoS One 14:e0219207. doi:10.1371/journal.pone.0219207. 31339899 PMC6655640

[18] Osaki H, Sasaki A, Nomiyama K, Tomioka K 2016 Multiple virus infection in a single strain of *Fusarium poae* shown by deep sequencing. Virus Genes 835–847. doi:10.1007/s11262-016-1379-x. 27550368

[19] Marzano S-YL, Nelson BD, Ajayi-Oyetunde O, Bradley CA, Hughes TJ, Hartman GL, Eastburn DM, Domier LL 2016 Identification of diverse mycoviruses through metatranscriptomics. J Virol 90:6846–6863. doi:10.1128/JVI.00357-16. 27194764 PMC4944287

[20] Sutela S, Poimala A, Vainio EJ 2019 Viruses of fungi and oomycetes in the soil environment. FEMS Microbiol Ecol 95:fiz119. doi:10.1093/femsec/fiz119. 31365065

[21] Bonfante P, Desirò A 2017 Who lives in a fungus? the diversity, origins, and functions of fungal endobacteria living in Mucoromycota. ISME J 11:1727–1735. doi:10.1038/ismej.2017.21. 28387771 PMC5520026

[22] Bozarth RF 1972 Mycoviruses: a new dimension in microbiology. Environ Health Perspect 2:23–39. 4628853 10.1289/ehp.720223 PMC1474899

[23] Wolf YI, Kazlauskas D, Iranzo J, Lucía-Sanz A, Kuhn JH, Krupovic M, Dolja VV, Koonin EV 2018 Origins and evolution of the global RNA virome. mBio 9:1–31. doi:10.1128/mBio.02329-18. PMC6282212 30482837

[24] Nibert ML, Vong M, Fugate KK, Debat HJ 2018 Evidence for contemporary plant mitoviruses. Virology 518:14–24. doi:10.1016/j.virol.2018.02.005. 29438872 PMC6668999

[25] Göker M, Scheuner C, Klenk HP, Stielow JB, Menzel W 2011 Codivergence of mycoviruses with their hosts. PLoS One 6:e22252. doi:10.1371/journal.pone.0022252. 21829452 PMC3146478

[26] Márquez LM, Redman RS, Rodriguez RJ, Roossinck MJ, Redman S 2007 A virus in a fungus in a plant: three-way symbiosis required for thermal tolerance. Science 315:513–515. doi:10.1126/science.1136237. 17255511

[27] Frank AC, Wolfe KH 2009 Evolutionary capture of viral and plasmid DNA by yeast nuclear Chromosomes. Eukaryot Cell 8:1521–1531. doi:10.1128/EC.00110-09. 19666779 PMC2756859

[28] Taylor DJ, Bruenn J 2009 The evolution of novel fungal genes from non-retroviral RNA viruses. BMC Biol 7:88. doi:10.1186/1741-7007-7-88. 20021636 PMC2805616

[29] Liu H, Fu Y, Jiang D, Li G, Xie J, Cheng J, Peng Y, Ghabrial SA, Yi X 2010 Widespread horizontal gene transfer from double-stranded RNA viruses to eukaryotic nuclear genomes. J Virol 84:11876–11887. doi:10.1128/JVI.00955-10. 20810725 PMC2977895

[30] Okada R, Kiyota E, Moriyama H, Fukuhara T, Natsuaki T 2015 A simple and rapid method to purify viral dsRNA from plant and fungal tissue. J Gen Plant Pathol 81:103–107. doi:10.1007/s10327-014-0575-6.

[31] Hannon GJ 2010 FASTX-Toolkit. http://hannonlab.cshl.edu/fastx_toolkit.

[32] Grabherr MG, Haas BJ, Yassour M, Levin JZ, Thompson DA, Amit I, Adiconis X, Fan L, Raychowdhury R, Zeng Q, Chen Z, Mauceli E, Hacohen N, Gnirke A, Rhind N, di Palma F, Birren BW, Nusbaum C, Lindblad-Toh K, Friedman N, Regev A 2011 Full-length transcriptome assembly from RNA-seq data without a reference genome. Nat Biotechnol 29:644–652. doi:10.1038/nbt.1883. 21572440 PMC3571712

[33] Haas BJ, Papanicolaou A, Yassour M, Grabherr M, Blood PD, Bowden J, Couger MB, Eccles D, Li B, Lieber M, MacManes MD, Ott M, Orvis J, Pochet N, Strozzi F, Weeks N, Westerman R, William T, Dewey CN, Henschel R, LeDuc RD, Friedman N, Regev A 2013 De novo transcript sequence reconstruction from RNA-seq using the Trinity platform for reference generation and analysis. Nat Protoc 8:1494–1512. doi:10.1038/nprot.2013.084. 23845962 PMC3875132

[34] Wheeler TS, Eddy SR 2013 nhmmer: DNA homology search with profile HMMs. Bioinformatics 29:2487–2489. doi:10.1093/bioinformatics/btt403. 23842809 PMC3777106

[35] Ruby JG, Bellare P, DeRisi JL 2013 PRICE: software for the targeted assembly of components of (meta)genomic sequence data. G3 (Bethesda) 3:865–880. doi:10.1534/g3.113.005967. 23550143 PMC3656733

[36] MacManes MD 2014 On the optimal trimming of high-throughput mRNA sequence data. Front Genet 5:1–7. doi:10.3389/fgene.2014.00013. 24567737 PMC3908319

[37] Dobin A, Davis CA, Schlesinger F, Drenkow J, Zaleski C, Jha S, Batut P, Chaisson M, Gingeras TR 2013 STAR: ultrafast universal RNA-seq aligner. Bioinformatics 29:15–21. doi:10.1093/bioinformatics/bts635. 23104886 PMC3530905

[38] Sasai S, Tamura K, Tojo M, Herrero M-L, Hoshino T, Ohki ST, Mochizuki T 2018 A novel non-segmented double-stranded RNA virus from an Arctic isolate of *Pythium polare*. Virology 522:234–243. doi:10.1016/j.virol.2018.07.012. 30055514

[39] Katoh K, Standley DM 2013 MAFFT multiple sequence alignment software version 7: improvements in performance and usability. Mol Biol Evol 30:772–780. doi:10.1093/molbev/mst010. 23329690 PMC3603318

[40] Capella-Gutiérrez S, Silla-Martínez JM, Gabaldón T 2009 trimAl: a tool for automated alignment trimming in large-scale phylogenetic analyses. Bioinformatics 25:1972–1973. doi:10.1093/bioinformatics/btp348. 19505945 PMC2712344

[41] Darriba D, Taboada GL, Doallo R, Posada D 2011 Europe PMC Funders Group ProtTest 3: fast selection of best-fit models of protein evolution. Bioinformatics 27:1164–1165. doi:10.1093/bioinformatics/btr088. 21335321 PMC5215816

[42] Stamatakis A 2014 RAxML version 8: a tool for phylogenetic analysis and post-analysis of large phylogenies. Bioinformatics 30:1312–1313. doi:10.1093/bioinformatics/btu033. 24451623 PMC3998144

[43] Roossink MJ, Saha P, Wiley GB, Quan J, White JD, Lai H, Chavarria F, Shen G, Roe BA 2010 Ecogenomics: using massively parallel pyrosequencing to understand viral ecology. Mol Ecol 19:81–88. doi:10.1111/j.1365-294X.2009.04470.x. 20331772

