# Erratum for Myers et al., “Survey of Early-Diverging Lineages of Fungi Reveals Abundant and Diverse Mycoviruses”

**DOI:** 10.1128/mBio.02851-20

**Published:** 2020-12-15

**Authors:** J. M. Myers, A. E. Bonds, R. A. Clemons, N. A. Thapa, D. R. Simmons, D. Carter-House, J. Ortanez, P. Liu, A. Miralles-Durán, A. Desirò, J. E. Longcore, G. Bonito, J. E. Stajich, J. W. Spatafora, Y. Chang, L. M. Corrochano, A. Gryganskyi, I. V. Grigoriev, T. Y. James

**Affiliations:** a University of Michigan, Department of Ecology and Evolutionary Biology, Ann Arbor, Michigan, USA; b University of California—Riverside, Department of Microbiology and Plant Pathology, Riverside, California, USA; c University of Seville, Department of Genetics, Seville, Spain; d Michigan State University, Department of Plant Soil and Microbial Sciences, East Lansing, Michigan, USA; e University of Maine, School of Biology and Ecology, Orono, Maine, USA; f Oregon State University, Department of Botany and Plant Pathology, Corvallis, Oregon, USA; g L. F. Lambert Spawn Co., Coatesville, Pennsylvania, USA; h U.S. Department of Energy Joint Genome Institute, Lawrence Berkeley National Laboratory, Berkeley, California, USA; i University of California—Berkeley, Department of Plant and Microbial Biology, Berkeley, California, USA

## ERRATUM

Volume 11, no. 5, e02027-20, 2020, https://doi.org/10.1128/mBio.02027-20. In the originally published version, there were errors in the author affiliations. The affiliations should appear as shown above.

