# Supplementary material for: Survey of Early-Diverging Lineages of Fungi Reveals Abundant and Diverse Mycoviruses
Source: mBio. 2020 Sep 8;11(5):e02027-20. doi: 10.1128/mBio.02027-20 (PMC7482067; doi:10.1128/mBio.02027-20)
Supplement: TABLE S2 [file mBio.02027-20-st002.docx]

**Supplementary Table 2.** Transcriptomes retrieved from SRA database.

| **Isolate** | **SRR** | **Citation** |
| --- | --- | --- |
| *Absidia repens* NRRL 1336 | SRR7974509 | Mondo SJ et al., 2017 doi: 10.1038/ng.3859 |
| *Allomyces macrogynus* ATCC_38327 | SRR343045 | unpublished |
| *Amylomyces rouxii* NRRL 5866 | SRR8529687 | unpublished |
| *Anaeromyces* sp. NHY-2018 | SRR7819341 | unpublished |
| *Anaeromyces* sp. S4 | SRR4063399 | Haitjema CH et al., 2017 doi: 10.1038/nmicrobiol.2017.87 |
| *Batrachochytrium dendrobatidis* 423 | SRR2719455 | Ellison, AR et al., 2017 doi: 10.1534/g3.116.035873 |
| *Batrachochytrium dendrobatidis* CLFT044 | SRR5988742 | McDonald, CA et al., 2019 doi: 10.1016/j.funbio.2019.10.008 |
| *Batrachochytrium salamandrivorans* | SRR3706726 | Farrer, RA et al. 2017 doi: 10.1038/ncomms14742 |
| *Benjaminiella poitrasii* RSA 903 | SRR6942796 | Unpublished |
| *Blakeslea trispora* F986 | SRR8238938 | Unpublished |
| *Blakeslea trispora* NRRL 2456 | SRR6049667 | Unpublished |
| *Blastocladiella britanica* JEL0711 | SRR6057017 | Unpublished |
| *Catenaria* sp. PL171 | SRR424218 | Mondo SJ et al., 2017 doi: 10.1038/ng.3859 |
| *Chaetocladium brefeldii* NRRL 2343 | SRR7975436 | Unpublished |
| *Chlamydoabsidia padenii* NRRL 2977 | SRR6057190 | Unpublished |
| *Choanephora cucurbitarum* NRRL 2744 | SRR8238940 | Unpublished |
| *Chytridium lagenaria* Arg66 | SRR6057011 | Unpublished |
| *Chytriomyces hyalinus* JEL0632 | SRR8189667 | Unpublished |
| *Chytriomyces* sp. nov. MP71 | SRR6056998 | Unpublished |
| *Claroideoglomus etunicatum* | DRR041158 | Unpublished |
| *Coelomomyces lativattus* CIRM-AVA-1 | SRR5504042 | Ahrendt, SA. 2015 ProQuest ID: Ahrendt_ucr_0032D_12303 |
| *Coemansia mojavensis* RSA 71 | SRR7140835 | Unpublished |
| *Coemansia spiralis* RSA 1278 | SRR6056689 | Unpublished |
| *Cokeromyces recurvatus* NRRL 2243 | SRR6056974 | Unpublished |
| *Conidiobolus thromboides* FSU 785 | SRR4052359 | Unpublished |
| *Dichotomocladium elegans* RSA 919 | SRR6256436 | Unpublished |
| *Dissophora ornata* CBS 347.77 | SRR8238935 | Unpublished |
| *Entomophaga maimaga* ARSEF 7190 | SRR9001799 | Unpublished |
| *Entomophthora muscae* HHdFL130914-01 | SRR5506701 | Nibert, ML et al. 2019 doi: 10.3390/v11040351 |
| *Entophlyctis helioformis* JEL0805 | SRR6057018 | Unpublished |
| *Gaertneriomyces semiglobifer* Barr 43 | SRR6056997 | Unpublished |
| *Geosiphon pyriformis* | SRR6363035 | Unpublished |
| *Geranomyces variabilis* JEL 559 | SRR8534491 | Unpublished |
| *Gigaspora margarita* BEG34 | SRR1659851 | Salvioli, A 2010 doi: 10.1111/j.1462-2920.2010.02246.x |
| *Gigaspora rosea* | SRR1979254 | Tang, N 2016 doi: 10.3389/fmicb.2016.00233 |
| *Gilbertella persicaria* var. *persicaria* CBS 190.32 | SRR6056754 | Unpublished |
| *Globomyces pollinis-pini* Arg68 | SRR6057014 | unpublished |
| *Gongronella butleri* C1D | SRR8303828 | unpublished |
| *Gorgonomyces haynaldii* MP0057 | SRR8267450 | unpublished |
| *Halteromyces radiatus* CBS 162.75 T | SRR7476973 | unpublished |
| *Helicostylum pulchrum* RSA 2064 | SRR8240038 | unpublished |
| *Hesseltinella vesiculosa* NRRL 3301 | SRR4063264 | Mondo SJ et al., 2017 doi: 10.1038/ng.3859 |
| *Hyaloraphidium curvatum* JEL0383 | SRR7517569 | unpublished |
| *Kickxella alabastrina* RSA 675 | SRR6057250 | unpublished |
| *Kirkomyces cordense* RSA 1222 | SRR6943043 | unpublished |
| *Linderina pennispora* ATCC 12442/NRRL 2237 | SRR3439779 | Mondo SJ et al., 2017 doi: 10.1038/ng.3859 |
| *Lobosporangium transversale* NRRL 3116 | SRR8840873 | Mondo SJ et al., 2017 doi: 10.1038/ng.3859 |
| *Martensiomyces pterosporus* CBS 209.56 | SRR4125806 | unpublished |
| *Mortierella alpina* | SRR1638091 | Wang, L et al., 2011 doi: 10.1371/journal.pone.0028319 |
| *Mortierella elongata* (wildtype) | SRR6225696 | Uehling, J et al., 2017 doi: 10.1111/1462-2920.13669 |
| *Mortierella elongata* | SRR6225691 | Uehling, J et al., 2017 doi: 10.1111/1462-2920.13669 |
| *Mortierella gamsii* AM 1032 | SRR5506997 | Unpublished |
| *Mortierella humilis* PMI 1414 | SRR6256461 | Unpublished |
| *Mortierella minutissima* AD051 | SRR6256835 | Unpublished |
| *Mortierella multidivaricata* RSA 2152 | SRR6256450 | Unpublished |
| *Mortierella* nov. sp. GBAus 27b | SRR6256463 | Unpublished |
| *Mortierella selenospora* CBS 811.68 | SRR6256464 | Unpublished |
| *Mortierella verticillata* NRRL 6337 | SRR343048 | Unpublished |
| *Mucor circinelloides* | SRR9009727 | Navarro-Mendoza MI et al., 2019 doi: 10.1016/j.cub.2019.09.024 |
| *Mucor irregularis* C3B | SRR8992276 | Barata, RR et al., 2019 doi: 10.1128/MRA.00503-19 |
| *Mycotypha africana* NRRL 2978 | SRR6049697 | unpublished |
| *Neocallimastix californiae* | SRR5296032 | Solomon, KV et al., 2016 doi: 10.1126/science.aad1431 |
| *Neocallimastix frontalis* 27 | SRR6829473 | Gruninger, RJ et al., 2018 doi: 10.3389/fmicb.2018.01581 |
| *Obelidium* sp. JEL0802 | SRR5506997 | unpublished |
| *Operculomyces laminatus* JEL223 | SRR5579326 | unpublished |
| *Orpinomyces* C1A | SRR2033890 | Couger, MB et al., 2015 doi: 10.1186/s13068-015-0390-0 |
| *Orpinomyces joyonii* SG4 | SRR6829438 | Gruninger, RJ et al., 2018 doi: 10.3389/fmicb.2018.01581 |
| *Paraglomus brasilianum* | SRR5279411 | Beaudet, D et al., 2018 doi: 10.1093/dnares/dsx051 |
| *Paraphysoderma sedebokerense* JEL0821 | SRR6942797 | Unpublished |
| *Parasitella parasitica* NRRL 2501 | SRR8840827 | Unpublished |
| *Phascolomyces articulosus* RSA 2281 | SRR6056973 | Unpublished |
| *Phycomyces blakesleeanus* | SRR9002717 | unpublished |
| *Phycomyces nitens* S609 | SRR6256453 | unpublished |
| *Piptocephalis cylindrospora* RSA 2659 | SRR5506707 | Ahrendt, SR et al., 2018 doi:10.1038/s41564-018-0261-0 |
| *Piromyces rhizinflatus* YM600 | SRR6829441 | Gruninger, RJ et al., 2018 doi: 10.3389/fmicb.2018.01581 |
| *Piromyces* *finn* | SRR5487626 | Haitjema CH et al., 2017 doi: 10.1038/nmicrobiol.2017.87 |
| *Polychytrium aggregatum* JEL109 | SRR9001797 | Unpublished |
| *Powellomyces hirtus* BR81 | SRR7517594 | Unpublished |
| *Ramicandelaber brevisporus* CBS 109374 | SRR4125814 | Unpublished |
| *Rhizoclosmatium globosum* JEL800 | SRR5506698 | Mondo SJ et al., 2017 doi: 10.1038/ng.3859 |
| *Rhizophagus diaphanum* MUCL 43196 | SRR916888 | Tisserant E et al., 2013 doi: 10.1073/pnas.1313452110 |
| *Rhizophagus intraradices* | SRR915897 | Tisserant E et al., 2013 doi: 10.1073/pnas.1313452110 |
| *Rhizophagus irregularis* | SRR7828199 | Nuepane, A et al., 2018 doi:10.3390/v10120707 |
| *Rhizophagus* sp. strain HR1 | DRR017631 | Kikuchi, Y et al., 2014 doi: 10.1111/nph.12937 |
| *Rhizopus microsporus* | SRR4489400 | Lastovetsky OA, et al., 2016 doi:10.1073/pnas.1615148113 |
| *Rhizopus oryzae* | SRR2104505 | Unpublished |
| *Rhizopus stolonifer* | SRR8191390 | Petrasch A, et al., 2019 doi: 10.3389/fpls.2019.00223 |
| *Syncephalastrum racemosum* NRRL 2495 | SRR8843209 | Mondo SJ et al., 2017 doi: 10.1038/ng.3859 |
| *Syncephalis ​fuscata*​ S228 | SRR6053271 | unpublished |
| *Syncephalis plumigaleata* NRRL S24 | SRR6050220 | unpublished |
| *Triparticalcar arcticum* BR59 | SRR7517624 | unpublished |
| *Umbelopsis isabellina* AD 026 | SRR6942795 | unpublished |
| *Umbelopsis nana* TLT 204 | SRR6256849 | unpublished |
| *Umbelopsis ramanniana* AG | SRR5487448 | unpublished |
| *Zoophthora radicans* ATCC 208865/ARSEF 4784 | SRR8189665 | unpublished |
| *Zopfochytrium polystomum* WB228 | SRR7141106 | unpublished |
